# Establishment of a SUMO pathway related gene signature for predicting prognosis, chemotherapy response and investigating the role of EGR2 in bladder cancer

**DOI:** 10.7150/jca.96481

**Published:** 2024-05-20

**Authors:** Han Xiao, Xiaoan Huang, Heyi Chen, Ying Zheng, Weihui Liu, Jiabi Chen, Ran Wei, Ming Lin, Qingshui Wang, Wei Zhuang

**Affiliations:** 1 Department of Urology, The Second Affiliated Hospital of Fujian Medical University, Quanzhou 362000, Fujian, China.; 2 Department of Pathology, Fujian Medical University Union Hospital, Fuzhou 350001, Fujian, China.; 3 Department of Obstetrics, Fuzhou Second Hospital, Fuzhou 350000, Fujian, China.; 4 The Second Affiliated Hospital of Fujian University of Traditional Chinese Medical University Medicine, Fujian-Macao Science and Technology Cooperation Base of Traditional Chinese Medicine-Oriented Chronic Disease Prevention and Treatment, Innovation and Transformation Center, Fujian University of Traditional Chinese Medicine, Fuzhou 350001, Fujian, China.; 5 School of Medical and Health Engineering, Changzhou University, Changzhou 213164, Jiangsu, China.

**Keywords:** Bladder cancer, SUMO, EGR2, Chemotherapy response, Prognosis

## Abstract

**Background:** Bladder cancer is a prevalent malignancy with significant clinical implications. Small Ubiquitin-like Modifier (SUMO) pathway related genes (SPRG) have been implicated in the development and progression of cancer.

**Methods:** In this study, we conducted a comprehensive analysis of SPRG in bladder cancer. We analyzed gene expression and prognostic value of SPRG and developed a SPRG signature (SPRGS) prognostic model based on four genes (HDAC4, TRIM27, EGR2, and UBE2I) in bladder cancer. Furthermore, we investigated the relationship between SPRGS and genomic alterations, tumor microenvironment, chemotherapy response, and immunotherapy. Additionally, we identified EGR2 as a key SPRG in bladder cancer. The expression of EGR2 in bladder cancer was detected by immunohistochemistry, and the cell function experiment clarified the effect of knocking down EGR2 on the proliferation, invasion, and migration of bladder cancer cells.

**Results:** Our findings suggest that SPRGS hold promise as prognostic markers and predictive biomarkers for chemotherapy response and immunotherapy efficacy in bladder cancer. The SPRGS prognostic model exhibited high predictive accuracy for bladder cancer patient survival. We also observed correlations between SPRG and genomic alterations, tumor microenvironment, and response to chemotherapy. Immunohistochemical results showed that EGR2 was highly expressed in bladder cancer tissues, and its overexpression was associated with poor prognosis. Knockdown of EGR2 inhibited bladder cancer cell proliferation, invasion, and migration.

**Conclusion:** This study provides valuable insights into the landscape of SPRGS in bladder cancer and their potential implications for personalized treatment strategies. The identification of EGR2 as a key SPRG and its functional impact on bladder cancer cells further highlights its significance in bladder cancer development and progression. Overall, SPRGS may serve as important prognostic markers and predictive biomarkers for bladder cancer patients, guiding treatment decisions and improving patient outcomes.

## Introduction

Bladder cancer (BLCA) is a common malignancy with significant morbidity and mortality worldwide 1. Despite advances in diagnosis and treatment, the prognosis for bladder cancer patients remains variable, highlighting the need for better prognostic markers and therapeutic targets. The Small Ubiquitin-like Modifier (SUMO) signaling pathway has emerged as a crucial regulator of various cellular processes, including DNA repair, transcriptional regulation, and protein stability^2^-^6^. Dysregulation of SUMO pathway-related genes (SPRG) have been implicated in the development and progression of several cancers, including bladder cancer^7^.

SUMOylation is a dynamic and reversible post-translational modification that regulates protein function at multiple levels^8^-^10^. So far, five subtypes of SUMO (SUMO1, 2, 3, 4, and 5) have been identified in the human genome. The enzyme cascade involved in regulating SUMOylation includes SUMO E1 activating enzyme, E2 conjugating enzyme, E3 ligase, and deSUMOylating enzymes. The SUMO pathway plays a crucial role in regulating various cellular processes associated with tumor development, such as cell cycle progression, stress response, hypoxia response, angiogenesis, invasion, stem cell-like properties, and immune response. In most published reports, the expression levels of SUMO pathway related genes are elevated in cancer and are associated with higher histological grade, advanced stage, presence of metastasis, and poor prognosis^11^-^14^. However, in certain types of cancer, high expression of SUMO pathway related genes is linked to a better prognosis^15^. Unfortunately, despite numerous studies highlighting the significance of SUMO pathway related genes in different types of tumors^16^-^19^, their specific roles in bladder cancer remain unclear, and there is a lack of relevant research. Therefore, our objective is to investigate the involvement of SUMO pathway related genes in bladder cancer.

In this study, we aimed to investigate the landscape of SPRG in bladder cancer and explore their potential prognostic and therapeutic implications. We first analyzed gene expression patterns of SUMO pathway-related genes in bladder cancer tissues compared to adjacent normal tissues. Subsequently, we examined the association between the expression levels of specific SPRG and bladder cancer prognosis. We also explored the genomic alterations and mutation frequencies of these genes in bladder cancer patients. Furthermore, we developed a SUMO pathway-related gene signature (SPRGS) prognostic model based on the expression levels of selected genes and evaluated its predictive accuracy for bladder cancer patient survival. Additionally, we investigated the potential role of SPRGS in predicting chemotherapy response in bladder cancer patients. Chemotherapy remains a key treatment modality for bladder cancer, but drug resistance poses a major challenge. Therefore, identifying predictive biomarkers for chemotherapy response is crucial for optimizing treatment outcomes. We analyzed the correlation between SPRGS expression and drug sensitivity, as well as the association between SPRGS and chemotherapy response in bladder cancer patients. Moreover, we explored the relationship between SPRGS and the tumor microenvironment and its potential implications for immunotherapy response. Furthermore, we focused on the functional significance of Early Growth Response 2 (EGR2), one of the identified SPRG associated with bladder cancer prognosis. We examined EGR2 expression in bladder cancer tissues and adjacent normal tissues and investigated its functional role in bladder cancer cell lines through knockdown experiments.

Overall, this study provides a comprehensive analysis of the landscape of SPRG in bladder cancer and explores their potential prognostic and therapeutic implications. The findings contribute to a better understanding of the molecular characteristics of bladder cancer and may facilitate the development of personalized treatment strategies for bladder cancer patients.

## Methods

### Data sources

To obtain the necessary data for our study, we downloaded transcriptome RNA-sequencing data along with clinical information pertaining to bladder cancer based on the TCGA-BLCA database and obtained the gene expression profiles and matched clinical information for the GSE13507, GSE32894, and GSE48075 cohorts from the Gene Expression Omnibus (GEO) database. The Programmed Death-Ligand 1 (PD-L1) treatment cohort for melanoma (Hugo cohort) were obtained from the TIDE database (http://tide.dfci.harvard.edu, Retrieved date: 2022.12.15). Human Gene Set (GO-BP protein SUMOylating) was obtained from Molecular Signatures Database (MSigDB, https://www.gsea-msigdb.org/gsea/msigdb, Retrieved date: 2022.10.15). The GO-BP protein SUMOylating set contains 68 genes, which are defined as SUMO pathway-related genes. The GSCA database (https://guolab.wchscu.cn/GSCA/#/) is a cancer genomics portal for gene set cancer analysis. In the study, it was used to analyze the relationship between genes expression and single nucleotide variation (SNV) in BLCA.

### Construction of a SUMO pathway-related gene signature (SPRGS)

To build a risk score module for SPRGS, we employed the transcriptome data from GSE13507 and utilized the LASSO Cox regression analysis. The genes with non-zero coefficients were defined as the final prognostic genes. These genes were used to construct a SPRGS model: SPRGS = (0.28 * expression of EGR2) + (0.77 * expression of Ubiquitin-Conjugating Enzyme E2I (UBE2I)) + (-0.33 * expression of Histone Deacetylase 4 (HDAC4)) + (-0.63 * expression of Tripartite Motif Containing 27 (TRIM27)).

### Chemotherapeutic and immune checkpoint Inhibitors (ICIs) immunotherapeutic response prediction

To identify potential molecular compounds for targeted therapy, we assessed the drug sensitivity of the SPRG genes. The Cancer Therapeutics Response Portal (CTRP) database (https://portals.broadinstitute.org/ctrp.v2.1/, Retrieved date: 2023.1.12) was employed for drug sensitivity analysis. Additionally, the Tumour Immune Dysfunction and Exclusion (TIDE) algorithm was utilized to predict ICIs that target the immunotherapeutic response based on RNA-Seq tumor expression profiles.

### Immune infiltration analysis

To estimate the immune infiltration in gastric cancer, we employed the CIBERSORT algorithm. We analyzed the correlation between SPRGS and immune cells.

### Cell culture

In this study, bladder cancer cell lines RT4 and T24 were obtained from American Type Culture Collection (ATCC). RT4 cells were cultured in DMEM medium, whereas T24 cells were cultured in McCoys 5A medium. Both cell types were supplemented with 1% penicillin-streptomycin and 10% fetal bovine serum. The cells were maintained in a humidified atmosphere with 5% CO_2_ at a temperature of 37°C.

### RT-PCR

RNA extraction was carried out using the Vazyme Total RNA Extraction Kit, and reverse transcription was performed using the TAKARA Reverse Transcription Kit. Subsequently, cDNA was synthesized and utilized as a template for PCR amplification, followed by qRT-PCR. The primer sequences are provided below: EGR2-F: 5'-CGGTGACCATCTTCCCCAAT-3'; EGR2-R: 5'-GAGCGAAGCTACTCGGATACG-3'; GAPDH-R: 5'-GCCATCACGCCACAGTTTC-3'; GAPDH-F: 5'-ACAACTTTGGTATCGTGGAAGG-3'.

### CCK-8 assay

RT4 and T24 cells were seeded into 96-well plates at a density of 2 x 10^4^ cells per well and cultured for 24, 48, and 72 hours. To measure cell viability, 10 µL of CCK-8 solution was added to each well four hours before absorbance measurement. Following a 2-hour incubation at 37°C, the absorbance was measured at 450 nm using a microplate reader.

### Invasion assay

RT4 and T24 cells were seeded in an invasion chamber using a serum-free medium. The lower chamber was filled with a complete medium and incubated at a temperature of 37°C with 5% CO_2_ for 48 hours. The cells that invaded and passed through the membrane were fixed using methanol and stained with crystal violet for a duration of 10 minutes. Cells present on the upper surface of the chamber were gently wiped off using cotton swabs, and the number of invasive tumor cells was randomly captured at six different locations.

### Migration assay

RT4 and T24 cells were seeded in six-well plates at a density of 1×10^6^ cells and placed in a constant temperature incubator for continued culture. When the cells reached at least 95% confluence, the six-well plates were marked using a 10µl gun tip. The marked area was washed three times with an appropriate amount of PBS, followed by the addition of 2 ml of serum-free medium. The six-well plates were then observed and photographed using an inverted phase contrast microscope to record the results. After 48 hours of culture, the serum-free medium was removed from the six-well plates, washed three times with PBS, and 2 ml of serum-free medium was added again. The scratched area was observed for migration and photographed for comparison. To ensure accuracy, the experiment was repeated three times for each independent group.

### Patients and specimens

A total of 80 samples of bladder cancer tissue and 80 paired corresponding non-tumor normal tissues were collected from the second affiliated hospital of fujian medical university between October 2016 and February 2018. The study was conducted following the guidelines and regulations approved by the Research Ethics Committee of the second affiliated hospital of fujian medical university. All patients participating in the study provided written informed consent under an institutionally approved protocol.

### Immunohistochemistry (IHC) staining analysis

IHC staining analysis was performed to evaluate the protein expression of EGR2 in both normal bladder tissue and bladder cancer tissue using the standard immunoperoxidase staining procedure. The slides were incubated with an anti-EGR2 antibody (13491-1-AP, Proteintech, Wuhan, China) at a dilution of 1:200. Two pathologists independently assessed the IHC staining scores for EGR2. Based on the percentage of positive cells in the field of view compared to the total number of cells, scoring is as follows: <1% positive cells are scored as 0, 1%-25% is scored as 1, 26%-50% is scored as 2, 51%-75% is scored as 3, and >76% is scored as 4. The intensity of positive cell staining is graded as follows: colorless is scored as 0, pale yellow is scored as 1, light brown is scored as 2, dark brown is scored as 3. The scores of both factors are multiplied to determine the positive grade: 0 is negative, 1-4 is weakly positive, 5-8 is positive, and 9-12 is strongly positive. Negative and weakly positive fall into the low expression group, while positive and strongly positive fall into the high expression group.

### Statistical analysis

The results are presented as mean ± SD and were compared using Student's t-test^20^, ^21^. Two-way ANOVA was employed to analyze the CCK8 assay^22^. A p-value less than 0.05 was considered statistically significant.

## Results

### The landscape of SUMO pathway-related gene (SPRG) in bladder cancer

**Figure 1:**
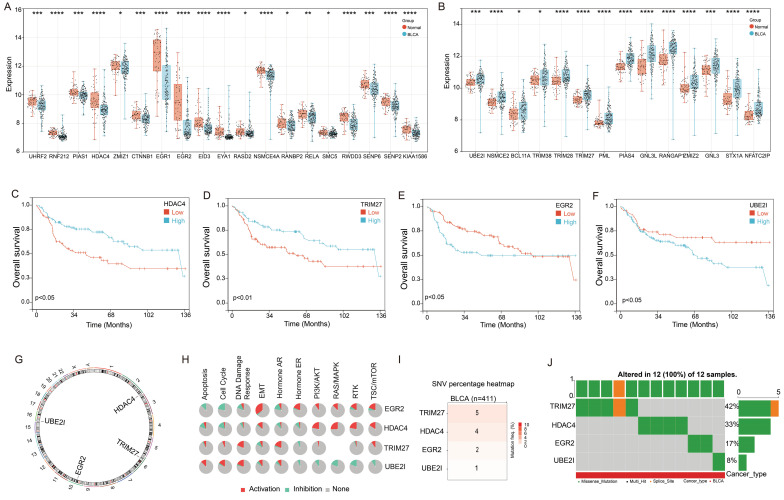
** Landscape of genetic variation and correlation of SPRG in bladder cancer.** (A) The downregulated and (B) upregulated SPRG in bladder cancer and normal bladder tissues based on GSE13507 cohort; The survival analysis of (C) HDAC4, (D)TRIM27, (E) EGR2, (F) UBE2I in bladder cancer based on GSE13507 cohort. (G) The chromosomal locations of the 4 genes were determined and their distribution was analyzed. (H) The associations between the 4 genes and relevant biological pathways. (I) The number of mutations present in 4 genes in cases of bladder cancer using TCGA-BLCA cohort. (J) Oncoplots showing the mutation landscape of 4 genes in bladder cancer patients from TCGA-BLCA cohort. *, *p*<0.05; **, *p*<0.01; ***, *p*<0.001; ****, *p*<0.0001.

First, we conducted an analysis of the 68 Small Ubiquitin-like Modifier (SUMO) signaling pathway gene expression in the bladder cancer. Utilizing the GSE13507 cohort, we observed significant downregulation of 19 genes and upregulation of 14 genes in bladder cancer tissues compared to adjacent tissues (Figure 1A&B). Moreover, we proceeded to explore the prognostic implications of these genes in bladder cancer. Our findings demonstrated that four genes, HDAC4, TRIM27, EGR2, and UBE2I, exhibited significant associations with bladder cancer prognosis. Notably, high expression levels of HDAC4 and TRIM27 were identified as favorable prognostic factors for bladder cancer patients, while elevated expression of EGR2 and UBE2I was associated with an unfavorable prognosis (Figure 1C-F).

Further examination revealed that HDAC4, TRIM27, EGR2, and UBE2I are all located on autosomes, with HDAC4 on chromosome 2, TRIM27 on chromosome 6, EGR2 on chromosome 10, and UBE2I on chromosome 16 (Figure 1G). Functional analysis results indicated that EGR2 displayed positive correlations with Epithelial-Mesenchymal Transition (EMT), Hormone Estrogen Receptor (ER), Phosphoinositide 3-Kinase/Protein Kinase B (PI3K/AKT), Rat Sarcoma/Mitogen-Activated Protein Kinase (RAS/MAPK), Receptor Tyrosine Kinase (RTK), and Tuberous Sclerosis Complex/Mammalian Target of Rapamycin (TSC/mTOR) signaling pathways, while exhibiting negative correlations with Apoptosis and cell cycle signaling pathways. HDAC4 primarily exhibited positive correlations with PI3K/AKT, RAS/MAPK, RTK, and TSC/mTOR signaling pathways. TRIM27 showed a significant positive correlation with the DNA Damage response signaling pathway, whereas UBE2I displayed negative correlations with Hormone ER, PI3K/AKT, RAS/MAPK, RTK, and TSC/mTOR signaling pathways, but positive correlations with Apoptosis, cell cycle, and DNA Damage response signaling pathways (Figure 1H).

In addition, we conducted an analysis of gene mutations in these four genes using the TCGA-BLCA cohort. The results indicated that among 411 cases of bladder cancer, TRIM27 gene mutations were detected in 5 patients, HDAC4 gene mutations in 4 patients, EGR2 mutations in 2 patients, and UBE2I gene mutations in 1 patient (Figure 1I). Notably, these 12 mutations occurred in different individuals diagnosed with bladder cancer (Figure 1J). This suggests that HDAC4, TRIM27, EGR2, and UBE2I exhibit a relatively low mutation rate in bladder cancer.

### Establishment of a SUMO pathway related gene signature (SPRGS) prognostic model for bladder cancer patients

**Figure 2:**
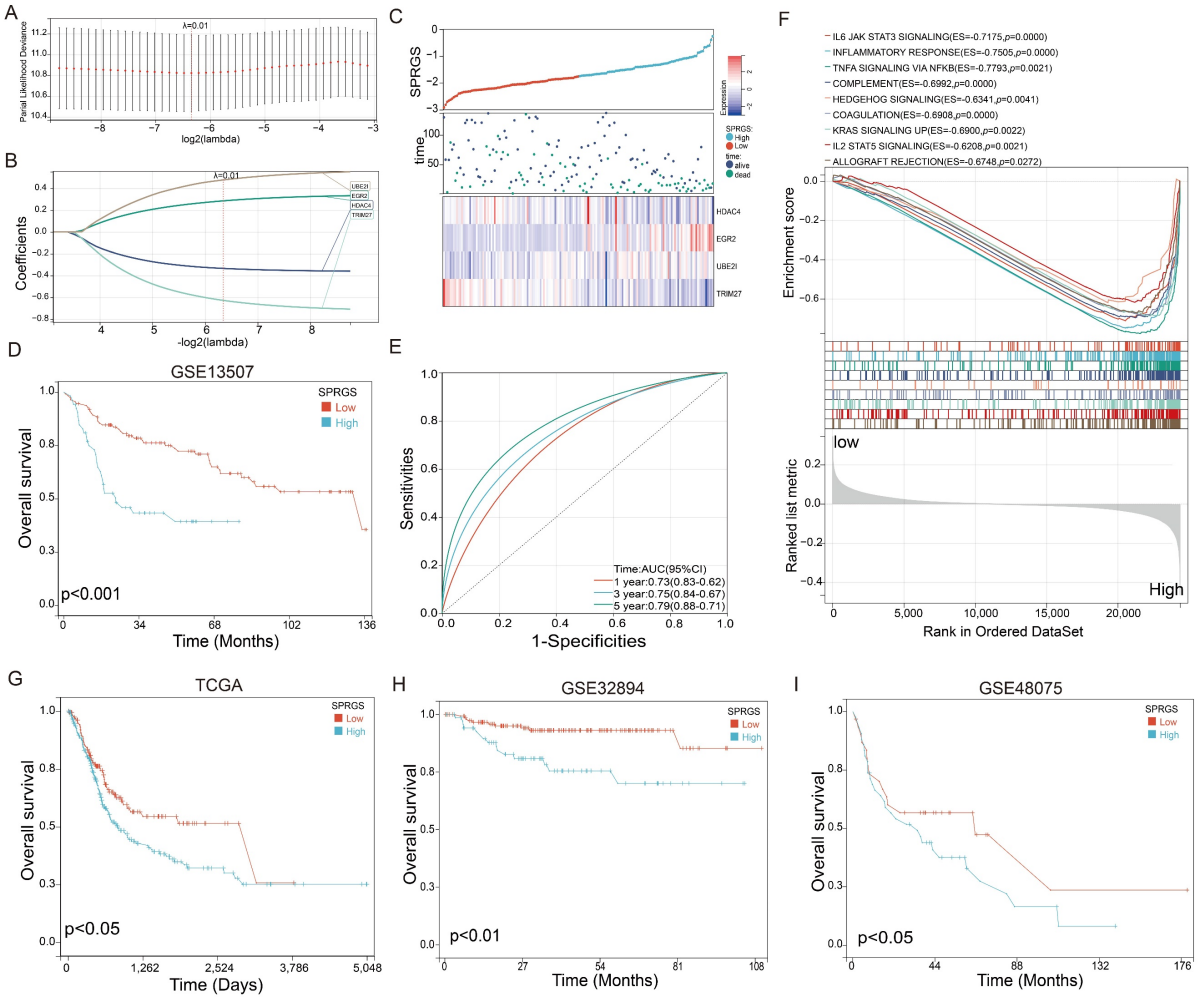
** Construction of SPRGS model to predict prognosis of bladder cancer patients.** (A) Lasso coefficient profile of overall survival with partial likelihood deviation; (B) Distribution of lasso coefficients for the SPRGS; (C) Distribution of SPRGS, survival status, and gene expression in patients based on the GSE13507 cohort; (D) The prognosis analysis of the SPRGS in GSE13507 cohort; (E) Time-dependent ROC analysis of the SPRGS; (F) Hallmark enrichment in the SPRGS-low and SPRGS-high subtypes; (G-I) The prognosis analysis of the SPRGS in (G) TCGA-BLCA, (H) GSE32894, and (I) GSE48075 cohorts. Samples were classified into SPRGS-low and SPRGS-high subtypes according to the best cut-off value for survival analysis.

To enhance the accuracy of prognostic prediction, we developed a LASSO Cox regression model and constructed a SUMO pathway-related gene signature (SPRGS) based on the expression levels of HDAC4, TRIM27, EGR2, and UBE2I. The process of constructing the SPRGS model is depicted in Figures 2A and 2B. Our 4-gene SPRGS prognostic model was established as follows: SPRGS = (0.28 * expression of EGR2) + (0.77 * expression of UBE2I) + (-0.33 * expression of HDAC4) + (-0.63 * expression of TRIM27). In Figure 2C, we present the expression levels of HDAC4, TRIM27, EGR2, and UBE2I, along with the survival status and survival time of the SPRGS-Low and SPRGS-High subtypes. The survival curve clearly indicates that patients in the SPRGS-High subtype exhibited significantly lower overall survival rates compared to those in the SPRGS-Low subtype (Figure 2D). Time receiver operating characteristic (ROC) analysis demonstrated that the SPRGS exhibited area under the curve (AUC) values of 0.73 for predicting one-year prognosis, 0.75 for three-year prognosis, and 0.79 for five-year prognosis in bladder cancer patients (Figure 2E). The hallmark enrichment analysis results demonstrated a significant enrichment of cancer-related gene sets, including IL6 (Interleukin 6)/JAK (Janus Kinase)/STAT3 (Signal Transducer and Activator of Transcription 3) signaling, Inflammatory response, Tumor Necrosis Factor-alpha (TNF-α) signaling via Nuclear Factor kappa-light-chain-enhancer of activated B cells (NF-kB), complement, hedgehog signaling, coagulation, KRAS signaling up, IL2/STAT5 signaling and allograft rejection the in the SPRGS-high subtype (Figure 2F). Consequently, targeting these pathways holds promising therapeutic potential for patients with the SPRGS-high subtype of bladder cancer patients.

Furthermore, we utilized the TCGA-BLCA, GSE32894 and GSE48075 cohorts as validation sets to analyze the prognostic value of SPRGS in bladder cancer. The results revealed that in the TCGA-BLCA, GSE32894 and GSE48075 cohorts, patients in the SPRGS-Low subtype had significantly better prognosis compared to patients in the SPRGS-High subtype (Figure 2G-I). This validation further supports the potential clinical utility of the SPRGS as a prognostic indicator in bladder cancer.

### Characterization of Molecular Features of SPRGS-Low and SPRGS-High Subtypes

**Figure 3:**
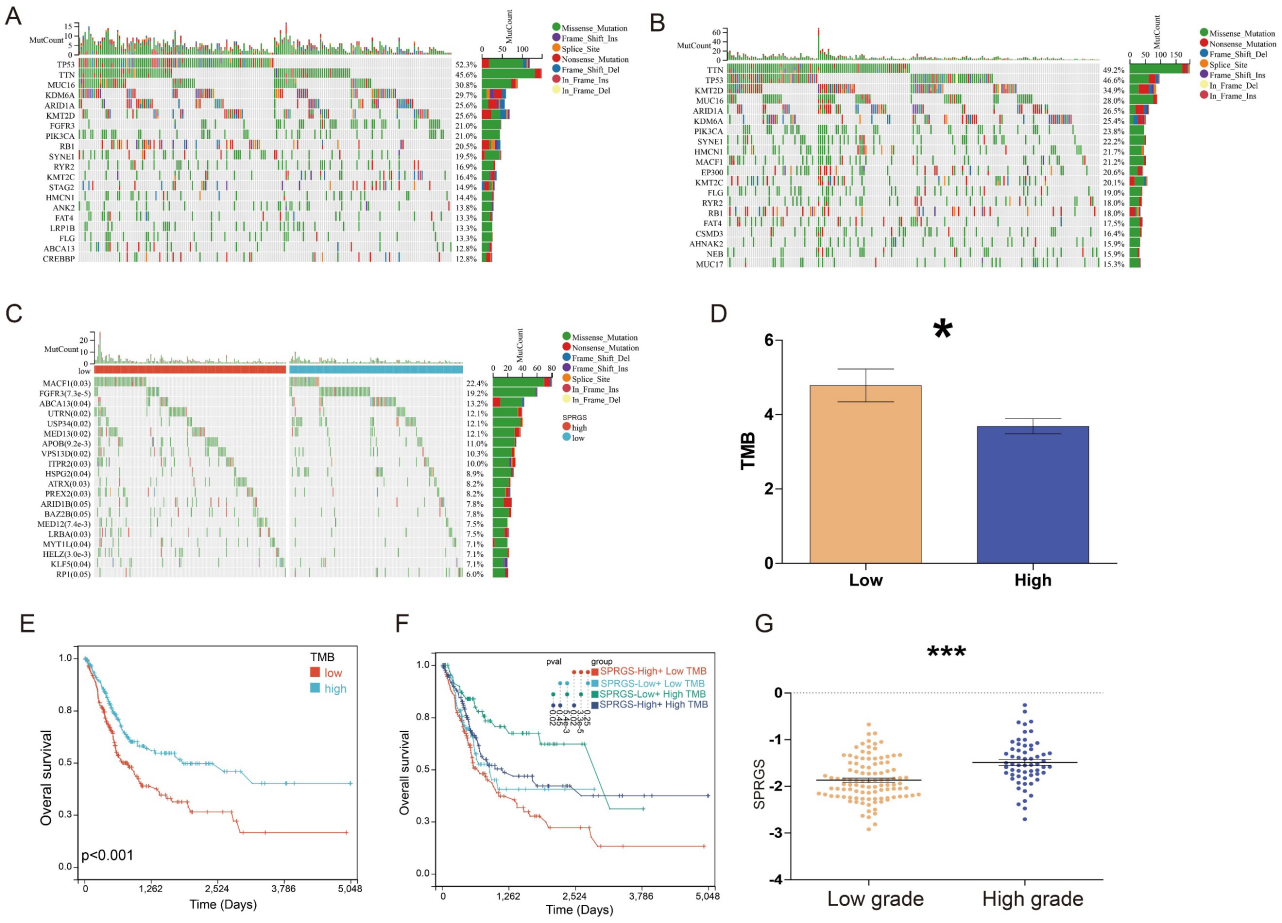
** Relationship among the SPRGS, genomic alterations, and molecular subtypes in bladder cancer.** Oncoplots showing landscapes of genomic alterations in (A) SPRGS-low and (B) SPRGS-high subtypes, respectively; (C) Top 20 genes with the highest mutation frequency related to the SPRGS based on TCGA-BLCA cohort; (D) Differences in tumor mutation burden between SPRGS-low and SPRGS-high subtypes; (E) The survival analysis of TMB in bladder cancer in TCGA-BLCA cohort; (F) The survival analysis of TMB and SPRGS in bladder cancer in TCGA-BLCA cohort; (G) The correlations between the SPRGS and different grade. *, *p*<0.05; ***, *p*<0.001.

We further conducted a genomic alteration analysis on the SPRGS-Low and SPRGS-High subtypes and noted that the top 20 genes with the highest mutation frequency exhibited a general similarity between the SPRGS-Low and SPRGS-High subtypes (Figure 3A&B). In the SPRGS-Low subtype, the top twenty genes with the highest mutation rates are Tumor Protein P53 (TP53) (52.3%), Titin (TTN) (45.6%), Mucin 16, Cell Surface Associated (MUC16) (30.8%), Lysine Demethylase 6A (KDM6A) (29.7%), AT-rich Interaction Domain 1A (ARID1A) (25.6%), Lysine Methyltransferase 2D (KMT2D) (25.6%), Fibroblast Growth Factor Receptor 3 (FGFR3) (21.0%), Phosphatidylinositol-4,5-Bisphosphate 3-Kinase Catalytic Subunit Alpha (PIK3CA) (21.0%), Retinoblastoma 1 (RB1) (20.5%), Spectrin Repeat Containing, Nuclear Envelope 1 (SYNE1) (19.5%), Ryanodine Receptor 2 (RYR2) (16.9%), Lysine Methyltransferase 2C (KMT2C) (16.4%), Stromal Antigen 2 (STAG2) (14.9%), Hemicentin 1 (HMCN1) (14.4%), Ankyrin 2 (ANK2) (13.8%), FAT Atypical Cadherin 4 (FAT4) (13.3%), Low Density Lipoprotein Receptor-Related Protein 1B (LRP1B) (13.3%), Filaggrin (FLG) (13.3%), ATP Binding Cassette Subfamily A Member 13 (ABCA13) (12.8%), and CREB Binding Protein (CREBBP) (12.8%). In the SPRGS-High subtype, the top twenty genes with the highest mutation rates include Titin (TTN) (49.2%), Tumor Protein P53 (TP53) (46.6%), Lysine Methyltransferase 2D (KMT2D) (34.9%), Mucin 16, Cell Surface Associated (MUC16) (28.0%), AT-rich Interaction Domain 1A (ARID1A) (26.5%), Lysine Demethylase 6A (KDM6A) (25.4%), Phosphatidylinositol-4,5-Bisphosphate 3-Kinase Catalytic Subunit Alpha (PIK3CA) (23.8%), Spectrin Repeat Containing, Nuclear Envelope 1 (SYNE1) (22.2%), Hemicentin 1 (HMCN1) (21.7%), Microtubule-Actin Crosslinking Factor 1 (MACF1) (21.2%), E1A Binding Protein P300 (EP300) (20.6%), Lysine Methyltransferase 2C (KMT2C) (20.1%), Filaggrin (FLG) (19.0%), Ryanodine Receptor 2 (RYR2) (18.0%), Retinoblastoma 1 (RB1) (18.0%), FAT Atypical Cadherin 4 (FAT4) (17.5%), CUB and Sushi Multiple Domains 3 (CSMD3) (16.4%), AHNAK Nucleoprotein 2 (AHNAK2) (15.9%), Nebulin (NEB) (15.9%), and Mucin 17, Cell Surface Associated (MUC17) (15.9%).

The significantly different mutated genes between the SPRGS-Low and SPRGS-High subtypes are depicted in Figure 3C. Moreover, tumor mutation burden (TMB) has emerged as a promising biomarker for immunotherapy due to its association with the production of immunogenic neoantigens. To delve deeper into this, we conducted an analysis to examine the disparity in TMB between the SPRGS-Low and SPRGS-High subtypes. Intriguingly, our findings revealed that the SPRGS-Low subtype exhibited a significantly higher level of TMB compared to the SPRGS-High subtype (Figure 3D). Survival analysis results further demonstrated a significant prognostic difference based on TMB status. Patients in the TMB-Low subgroup exhibited considerably better prognosis compared to those in the TMB-High subgroup (Figure 3E). Additionally, additional analysis shed light on the combined effect of SPRGS and TMB on patient survival. The SPRGS-High+TMB-Low subtype displayed the lowest survival rate, while the SPRGS-Low+TMB-High subtype exhibited the highest survival rate (Figure 3F). These findings highlight the potential clinical relevance of considering both SPRGS and TMB as prognostic factors in bladder cancer.

Furthermore, we extended our analysis to explore the association between SPRGS and different grades of bladder cancer. The results revealed a significant difference in SPRGS expression between high-grade and low-grade patients, with high-grade individuals displaying notably higher SPRGS levels (Figure 3G). This suggests that SPRGS may play a role in the progression and aggressiveness of bladder cancer.

### SPRGS predicted the response to chemotherapy in bladder cancer

**Figure 4:**
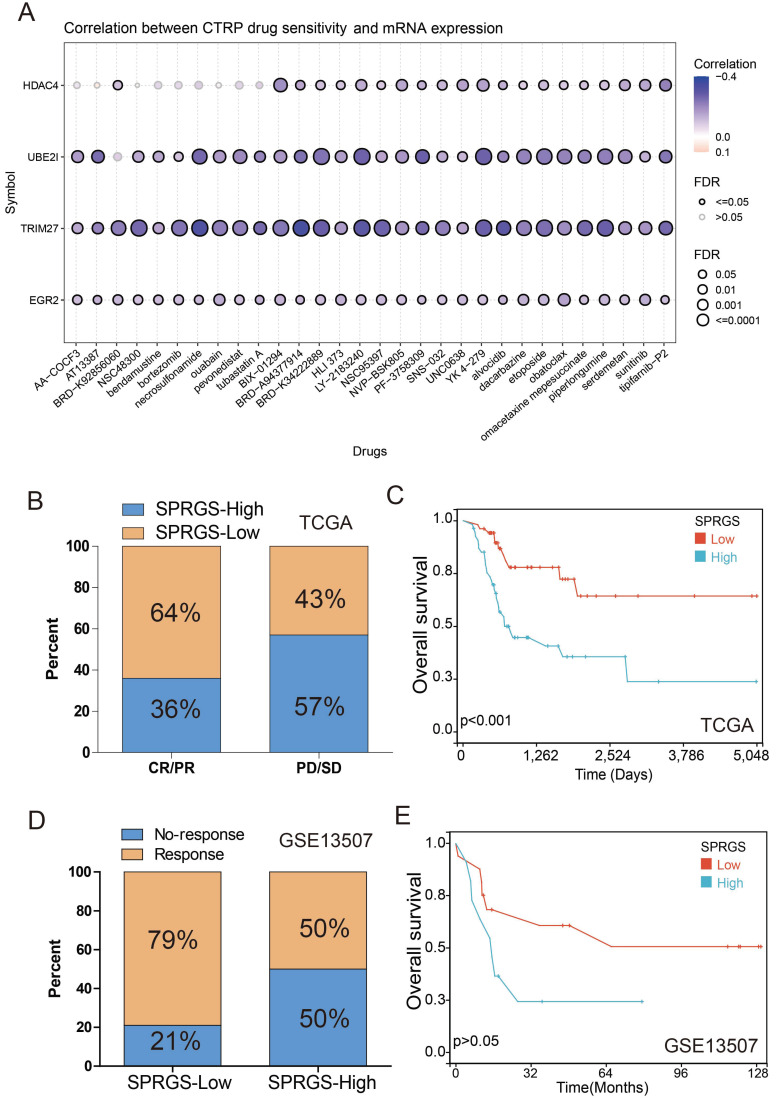
** Prediction and correlation of the sensitivity to chemotherapy drugs in bladder cancer**. (A) The correlation between GDSC drug sensitivity and 4 genes expression; (B) The correlation of SPRGS with response to chemotherapy in TCGA-BLCA cohort; (C) The predictive value of SPRGS in bladder cancer patients treated with chemotherapy in TCGA-BLCA cohort; (D) The correlation of SPRGS with response to chemotherapy in GSE13507 cohort; (E) The predictive value of SPRGS in bladder cancer patients treated with chemotherapy in GSE13507 cohort. Samples were classified into SPRGS-low and SPRGS-high subtypes according to the best cut-off value for survival analysis.

Multiple clinical studies have consistently demonstrated that chemotherapy can significantly improve the prognosis of bladder cancer patients in comparison to surgery alone^23^-^26^. However, the development of drug resistance remains a major hurdle in achieving successful chemotherapy outcomes. To address this challenge, we investigated the correlation between drug sensitivity and SPRGS gene expression using data from the Cancer Therapeutics Response Portal (CTRP) database. Remarkably, our findings revealed a negative correlation between the expression levels of HDAC4, UBE2I, TRIM27, EGR2 genes, and the IC50 values of various anticancer drugs (Figure 4A). These important insights can guide the development of more effective chemotherapy regimens tailored to individual patients.

Previous studies have indicated that somatic mutations in FGFR3 in bladder cancer patients could serve as potential predictive biomarkers for neoadjuvant chemotherapy response^27^. This study found a significantly higher mutation rate of FGFR3 in the SPRGS-Low subtype compared to the SPRGS-High subtype (Figure 3C), suggesting that SPRGS may be a potential predictive biomarker for chemotherapy response. Therefore, we conducted a comprehensive analysis to explore the relationship between SPRGS and chemotherapy response among a cohort of 109 bladder cancer patients who underwent chemotherapy, utilizing the TCGA cohort. Intriguingly, our results demonstrated that 64% of patients in the complete or partial response (CR/PR) group belonged to the SPRGS-Low subtype, while 36% belonged to the SPRGS-High subtype. In contrast, among patients in the progressive disease or stable disease (PD/SD) group, 43% belonged to the SPRGS-Low subtype, while 57% were classified as SPRGS-High subtype (Figure 4B). Furthermore, patients in the SPRGS-Low subtype exhibited a significantly more favorable overall survival rate compared to those in the SPRGS-High subtype (Figure 4C). These findings highlight the potential of SPRGS as a predictive biomarker for chemotherapy response and prognosis in bladder cancer patients.

Additionally, we analyzed data from the GSE13507 cohort, which included 27 bladder cancer patients who received systemic chemotherapy. Within the SPRGS-Low subtype, an impressive 79% of bladder cancer patients responded positively to chemotherapy, while in the SPRGS-High subtype, the response rate was 50% (Figure 4D). Survival analysis further revealed that patients in the SPRGS-Low subtype had a notably superior overall survival rate compared to those in the SPRGS-High subtype (Figure 4E). However, it is important to note that these results did not reach statistical significance, likely due to the relatively small sample size. Nonetheless, these findings underscore the potential utility of SPRGS in predicting chemotherapy response and informing treatment decisions for bladder cancer patients.

### The relation between SPRGS and tumor microenvironment and immunotherapy

**Figure 5:**
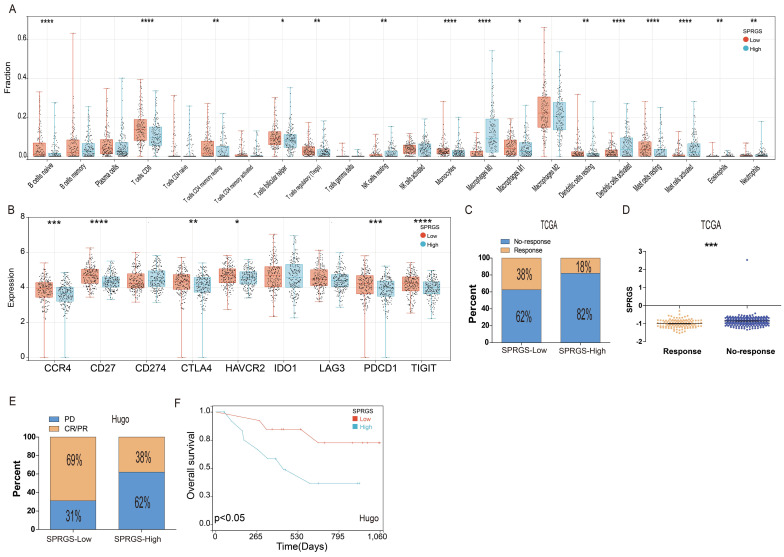
** Correlation between the SPRGS and tumor microenvironment and immunotherapy in bladder cancer.** (A) Box plots illustrating the relationships between SPRGS subtypes and the infiltration of immune cells. (B) Box plots illustrating the relationships between SPRGS subtypes and the immune checkpoint genes; (C) The correlation of SPRGS with response to chemotherapy in TCGA-BLCA cohort; (D) The value the SPRGS in response and No-response group; (E) The correlation of SPRGS with response to chemotherapy in Hugo cohort; (F) The predictive value of SPRGS in bladder cancer patients treated with immunotherapy in Hugo cohort. Samples were classified into SPRGS-low and SPRGS-high subtypes according to the best cut-off value for survival analysis. ***, *p*<0.001.

To investigate the interplay between the SPRGS and the immune microenvironment, we conducted an analysis of immune cell infiltration in both the SPRGS-low and SPRGS-high subtypes. The tumor microenvironment is recognized for its profound influence on tumor progression and therapeutic response. Our findings reveal that the SPRGS-Low subtype exhibits elevated levels of infiltrating B cells native, T cells CD8, T cells CD4 memory resting, T cells follicular helper, T cells regulatory, Monocytes, Macrophages M1, Dendritic cells resting, Mast cells resting, and Eosinophils. Conversely, the SPRGS-High subtype demonstrates increased infiltration of NK cells resting, Macrophages M0, Dendritic cells activated, Mast cells activated, and Neutrophils (Figure 5A).

Furthermore, we explored the differential expression of common immune checkpoint genes between the SPRGS-Low and SPRGS-High subtypes. Notably, CCR4, CD27, CTLA4, HAVCR2, PDCD1, and TIGIT exhibited significantly higher expression in the SPRGS-Low subtype compared to the SPRGS-High subtype (Figure 5B). These findings shed light on the potential role of the immune microenvironment in shaping the distinct characteristics of the SPRGS subtypes.

To predict the likelihood of response to immune checkpoint inhibitor (ICI) therapy, we employed the TIDE algorithm utilizing transcriptome data. Intriguingly, our results indicate that the proportion of patients predicted to respond to immunotherapy was 38% in the SPRGS-Low subtype, while it was only 18% in the SPRGS-High subtype (Figure 5C). Importantly, patients who responded to immunotherapy displayed significantly lower SPRGS values compared to non-responders (Figure 5D). This suggests that the SPRGS may serve as a valuable tool for predicting the efficacy of immunotherapy in bladder cancer patients.

Additionally, we further assessed the predictive capability of the SPRGS model using the Hugo cohort. In the SPRGS-Low subtype, a remarkable 69% of patients achieved CR/PR, while 31% experienced PD. In contrast, among patients in the SPRGS-High subtype, the proportions of CR/PR and PD were 38% and 62%, respectively (Figure 5E). Survival analysis results demonstrated that patients in the SPRGS-Low subtype exhibited higher survival rates compared to those in the SPRGS-High subtype (Figure 5F). These findings underscore the potential of the SPRGS as a predictive biomarker for treatment response and prognosis in bladder cancer patients.

### High expression of EGR2 mRNA in bladder cancer is associated with poor prognosis

**Figure 6:**
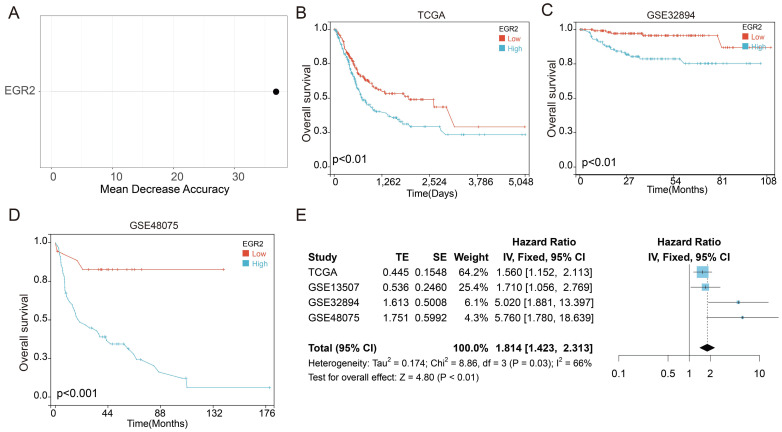
** High expression of EGR2 mRNA in bladder cancer is associated with poor prognosis.** (A) Random forest feature importance ranking for the SPRGS; (B-D) Prognostic analysis investigating the role of EGR2 in predicting outcomes within the (B) TCGA-BLCA, (C) GSE32894, and (D) GSE48975 cohorts. (E) The meta-analysis indicated that the bladder cancer patients with high EGR2 suffered poorer OS.

Next, we employed random forest analysis to assess the significance of the four SPRG in distinguishing between bladder cancer tissues and adjacent normal tissues. Through this analysis, we aimed to identify key genes associated with bladder cancer. Interestingly, our findings highlighted EGR2 as the most significant gene among the 4 SPRG genes (Figure 6A). In addition, we further analyzed the prognostic value of EGR2 in bladder cancer patients using the TCGA, GSE32894, and GSE48075 cohorts (Figure 6B-D). Next, we conducted an extensive meta-analysis incorporating both the TCGA, GSE32894, GSE13507, and GSE48075cohorts (Figure 6E). The results showed that low expression of EGR2 is a favorable prognostic factor for bladder cancer patients.

**Figure 7:**
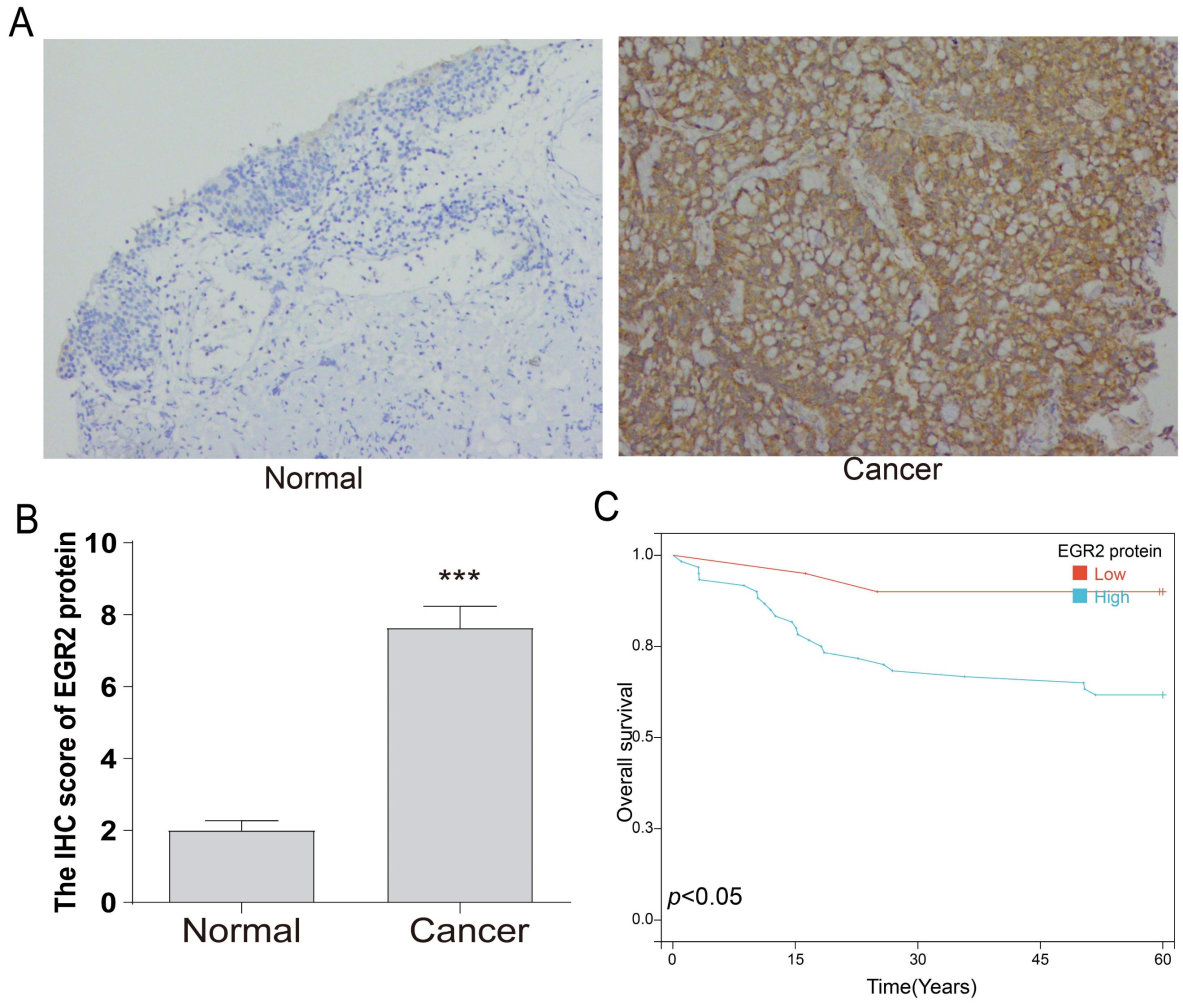
** High expression of EGR2 protein in bladder cancer is associated with poor prognosis.** (A) Representative IHC images of EGR2 protein in normal bladder and bladder cancer tissues. (B) Bar graph of the statistical analysis of A; (C) Survival analysis of EGR2 protein in bladder cancer. ***, *p*<0.001.

Following that, we conducted an in-depth investigation of EGR2 expression in bladder cancer through immunohistochemistry. We evaluate the expression of EGR2 protein in 80 paired bladder cancer samples, encompassing both cancerous and adjacent tissues. Our experimental findings unveiled a significant upregulation of EGR2 protein expression in bladder cancer tissues as compared to adjacent tissues (Figure 7A-B). Furthermore, our clinical correlation analysis revealed a correlation between low EGR2 expression and Stage T1&2 (Table 1). Survival analysis provided additional evidence that high expression of EGR2 protein is an unfavorable prognostic factor for patients with bladder cancer (Figure 7C).

### EGR2 Knockdown inhibits the proliferation, invasion and migration of bladder cancer cells

**Figure 8:**
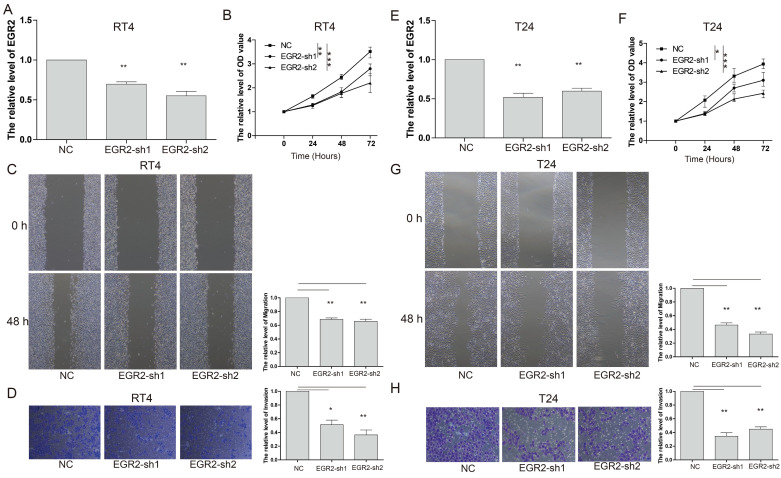
** Knockdown of EGR2 inhibits bladder cell proliferation migration, and invasion.** The EGR2 expression in the (A) RT4 and (B) T24 cells transfected with EGR2-shRNAs; Effect of knockdown EGR2 on (C) RT4 and (D) T24 growth determined by CCK-8 assay; Effect of reduced EGR2 expression on (E) RT4 and (F) T24 cell migration; Effect of reduced EGR2 expression on (G) RT4 and (H) T24 cell invasion. *, *p*<0.05; **, *p*<0.01; ***, *p*<0.001.

To gain further insights into the functional impact of EGR2 in bladder cancer cells, we conducted targeted experiments to knockdown EGR2 expression in RT4 and T24 bladder cancer cell lines. The objective was to evaluate the effects of EGR2 on crucial cellular processes such as proliferation, invasion, and migration. Our results from the CCK-8 assay demonstrated a significant inhibition of cell proliferation in RT4 cells when EGR2 expression was downregulated (Figure 8A&B). The scratch assay revealed a notable suppression of cell migration in RT4 cells with reduced EGR2 expression (Figure 8C). Additionally, the Transwell assay provided evidence of a significant inhibition of cell invasion in RT4 cells following EGR2 downregulation (Figure 8D). Similarly, EGR2 knockdown can significantly inhibit the proliferation, migration, and invasion abilities of T24 cells (Figure 8E-H). These findings collectively suggest that EGR2 plays a critical role in promoting cell proliferation, invasion, and migration in bladder cancer cells.

## Discussion

In this study, we conducted a comprehensive investigation of the landscape of SUMO pathway-related genes (SPRG) in bladder cancer. Our findings shed light on the potential prognostic value and functional significance of SPRG in bladder cancer. We identified four key genes, HDAC4, TRIM27, EGR2, and UBE2I, that exhibited significant associations with bladder cancer prognosis. Additionally, we explored the genomic alterations, tumor mutation burden (TMB), and chemotherapy response in relation to SPRG expression. Furthermore, we characterized the immune microenvironment and assessed the predictive potential of SPRG for immunotherapy response. Finally, we focused on the role of EGR2 in bladder cancer and demonstrated its impact on cell proliferation, invasion, and migration.

Our analysis revealed significant downregulation of 19 genes and upregulation of 14 genes related to the SUMO signaling pathway in bladder cancer tissues compared to adjacent tissues. Among these genes, HDAC4 and TRIM27 were associated with a favorable prognosis, while EGR2 and UBE2I were linked to an unfavorable prognosis.

HDAC4 is a member of the IIa class of the deacetylase family, alongside HDAC5, HDAC7, and HDAC9^28^. These epigenetic regulators control the acetylation/deacetylation cycles of lysine by counteracting histone acetyltransferases (HAT/KAT). Mutations and dysregulated expression of HDAC4 have been observed in various tumor types. Soft tissue sarcoma exhibits the highest mutation rate (>30%) for HDAC4, while non-small cell lung cancer and melanoma have the second highest frequency of HDAC4 gene alterations (15-17%)^28^. Elevated levels of HDAC4 mRNA are unfavorable prognostic markers in ovarian cancer but are considered favorable in pancreatic cancer^29^. The precise molecular mechanisms underlying HDAC4's role in cancer remain incompletely understood, but it may involve interactions with the corepressor complex NCOR1/NCOR2/HDAC3 and influence the transcription landscape of cancer cells through epigenomic reprogramming.

TRIM27 is a RING-mediated E3 ubiquitin ligase that marks other proteins for degradation^30^. Its targets for ubiquitination include PTEN, IκBα, and p53, allowing it to regulate multiple signaling pathways involved in cell proliferation, differentiation, and apoptosis. Abnormal expression of TRIM27 has been observed in various cancers, such as hepatocellular carcinoma, non-small cell lung cancer, ovarian cancer, and breast cancer^31^-^33^. High levels of TRIM27 expression in these cancer types are associated with unfavorable clinical features and a poor prognosis.

The EGR2 gene, also known as Early Growth Response 2 or Krox-20, is a transcription factor gene involved in regulating important biological processes such as cell proliferation, differentiation, and the cell cycle. In patients with advanced chronic lymphocytic leukemia, recurrent mutations within EGR2 have been associated with poor prognosis^34^. EGR2-mutated patients often carry ATM mutations (42%), TP53 alterations (18%), and NOTCH1/FBXW7 mutations (16%). In tumors, the role of the EGR2 gene can be dual. On one hand, EGR2 acts as a tumor-suppressive factor and exhibits anti-cancer effects in certain types of tumors. It is downregulated in liver cancer, and overexpression of EGR2 can inhibit the invasive ability of HepG2 liver cancer cells. Knockdown of EGR2 promotes gastric cancer cell growth and suppresses apoptosis. In thyroid cancer, overexpression of EGR2 can inhibit the progression of the disease. On the other hand, in certain circumstances, the EGR2 gene may also play a role in promoting cancer^35^, ^36^. For example, EGR2 can drive renal cancer occurrence and metastasis by enhancing S1PR3 mRNA stability^37^. Overall, the role of the EGR2 gene in tumor development and progression is complex and can vary depending on the specific context and tumor type. Further research is needed to gain a deeper understanding of the specific functions and potential therapeutic value of EGR2 in different types of cancers.

UBE2I, also known as UBC9, is a member of the E2 enzyme family and is involved in post-translational regulation of protein expression^38^, ^39^. Several studies have demonstrated that UBE2I plays a critical role in the development and progression of various cancers^40^-^42^. UBE2I is a key regulatory factor in the body's immune response. It is necessary for positive selection and late-stage maturation of thymocytes. UBE2I has also been found to have beneficial effects on cardiac function in damaged hearts through mediating SUMOylation. Moreover, aberrant expression of UBE2I has been implicated in the development and progression of various human cancers, including breast cancer, gliomas, lung cancer, head and neck squamous cell carcinoma, osteosarcoma, and hepatocellular carcinoma^43^.

The location of these genes on autosomes suggests their independent roles and potential distinct mechanisms of action in bladder cancer. Functional analysis revealed correlations between SPRG expression and various signaling pathways, including EMT, Hormone ER, PI3K/AKT, RAS/MAPK, RTK, TSC/mTOR, Apoptosis, cell cycle, and DNA Damage response pathways. These findings suggest that SPRG dysregulation may contribute to altered cellular processes and signaling pathways in bladder cancer. Gene mutation analysis indicated a relatively low mutation rate in HDAC4, TRIM27, EGR2, and UBE2I in bladder cancer. This implies that the dysregulation of these genes is primarily driven by alterations in gene expression rather than genetic mutations.

To improve prognostic prediction, we developed a SUMO pathway-related gene signature (SPRGS) based on the expression levels of HDAC4, TRIM27, EGR2, and UBE2I. The SPRGS model demonstrated significant prognostic value in distinguishing between SPRGS-Low and SPRGS-High subtypes. Patients in the SPRGS-High subtype had significantly lower overall survival rates compared to those in the SPRGS-Low subtype. The SPRGS also showed promise as a predictive model for one-year, three-year, and five-year prognosis in bladder cancer patients. Validation using the TCGA-BLCA cohort further supported the prognostic value of SPRGS in bladder cancer. Patients in the SPRGS-Low subtype had significantly better prognosis compared to those in the SPRGS-High subtype, confirming the potential clinical utility of SPRGS as a prognostic indicator.

The advancements in Next-Generation Sequencing (NGS) have revealed the human genomic landscape. Furthermore, the information generated by NGS has contributed to the development of precision oncology and personalized medicine. Previous studies have indicated that somatic mutations in FGFR3 in bladder cancer patients could serve as potential predictive biomarkers for neoadjuvant chemotherapy response^44^. This study found a significantly higher mutation rate of FGFR3 in the SPRGS-Low subtype compared to the SPRGS-High subtype, suggesting that SPRGS may be a potential predictive biomarker for chemotherapy response. The SPRGS also showed promise in predicting chemotherapy response, as patients in the SPRGS-Low subtype exhibited better prognosis and higher response rates to chemotherapy. Furthermore, we observed that SPRGS expression was associated with tumor grade, with high-grade bladder cancer patients displaying higher SPRGS levels.

Moreover, we investigated the association between the SPRGS and the tumor microenvironment, as well as the potential implications for immunotherapy response in bladder cancer. Our analysis revealed distinct differences in immune cell infiltration patterns between the SPRGS-Low and SPRGS-High subtypes. The SPRGS-Low subtype exhibited elevated levels of infiltrating B cells native, T cells CD8, T cells CD4 memory resting, T cells follicular helper, T cells regulatory, Monocytes, Macrophages M1, Dendritic cells resting, Mast cells resting, and Eosinophils. Conversely, the SPRGS-High subtype demonstrated increased infiltration of NK cells resting, Macrophages M0, Dendritic cells activated, Mast cells activated, and Neutrophils.

Furthermore, we explored the differential expression of common immune checkpoint genes between the SPRGS-Low and SPRGS-High subtypes. Notably, CCR4, CD27, CTLA4, HAVCR2, PDCD1, and TIGIT exhibited significantly higher expression in the SPRGS-Low subtype compared to the SPRGS-High subtype. These immune checkpoint genes play critical roles in regulating immune responses and may indicate a more immunologically active tumor microenvironment in the SPRGS-Low subtype. To predict the likelihood of response to immune checkpoint inhibitor (ICI) therapy, we utilized the TIDE algorithm and found that a higher proportion of patients in the SPRGS-Low subtype were predicted to respond to immunotherapy compared to the SPRGS-High subtype. Additionally, patients who responded to immunotherapy displayed significantly lower SPRGS values compared to non-responders. These findings suggest that the SPRGS may serve as a valuable tool for predicting the efficacy of immunotherapy in bladder cancer patients. To further validate the predictive capability of the SPRGS model, we assessed the treatment response and survival outcomes in the Hugo2016 cohort. The results demonstrated that a higher proportion of patients in the SPRGS-Low subtype achieved complete or partial response (CR/PR) to treatment, while a higher proportion of patients in the SPRGS-High subtype experienced progressive disease (PD). Additionally, patients in the SPRGS-Low subtype exhibited higher survival rates compared to those in the SPRGS-High subtype. These findings provide further support for the potential of the SPRGS as a predictive biomarker for treatment response and prognosis. Overall, our comprehensive analyses highlight that SPRGS holds promise as a predictive biomarker for immunotherapy response and may guide personalized treatment decisions in bladder cancer.

Finally, random forest analysis identified EGR2 as the most significant gene among the four SPRG examined, highlighting its potential importance in bladder cancer. Immunohistochemistry analysis further confirmed that EGR2 expression was substantially upregulated in bladder cancer tissues compared to adjacent normal tissues, suggesting a potential role for EGR2 in bladder cancer development or progression. To gain further insights into the functional impact of EGR2 in bladder cancer cells, we conducted targeted experiments to knock down EGR2 expression in RT4 and T24 bladder cancer cell lines. The results demonstrated that downregulation of EGR2 significantly inhibited cell proliferation, migration, and invasion in both RT4 and T24 cells. These findings indicate that EGR2 plays a critical role in promoting these crucial cellular processes in bladder cancer cells.

While our study provides valuable insights into the landscape of SPRG in bladder cancer and their potential prognostic and therapeutic implications, there are several limitations that should be acknowledged. First, our study primarily relied on publicly available datasets, such as the GSE13507 cohort and the TCGA bladder cancer dataset. Although these datasets provide a large amount of data for analysis, they may still have inherent biases and limitations. The samples included in these datasets may not fully represent the entire bladder cancer population, and there may be variations in data quality and experimental procedures across different studies. Therefore, caution should be exercised when generalizing our findings to the broader bladder cancer patient population. Second, the functional role of SPRGs in bladder cancer was primarily investigated through in vitro experiments using cell lines. While these experiments provide valuable insights into the potential impact of SPRGs on cellular processes, they may not fully recapitulate the complex tumor microenvironment and heterogeneity present in actual patient tumors. Further in vivo experiments and studies using patient-derived xenograft models would be necessary to validate and expand upon our findings. Third, our study focused on the prognostic and therapeutic implications of SPRGs in bladder cancer. However, the underlying molecular mechanisms and pathways through which these genes exert their effects were not fully elucidated. Further functional studies, such as gene knockdown or overexpression experiments, pathway analysis, and mechanistic investigations, are needed to better understand the biological significance of SPRGs in bladder cancer. Lastly, our study primarily focused on the SPRGs identified in the SUMO signaling pathway. While these genes have shown promising associations with bladder cancer prognosis and therapeutic response, it is important to acknowledge that other genes and pathways may also contribute to the overall landscape and heterogeneity of bladder cancer. Future studies should consider a more comprehensive analysis of the bladder cancer genome and transcriptome to capture the full complexity of the disease.

## Conclusion

In conclusion, our study provides a comprehensive analysis of the landscape of SUMO pathway-related genes (SPRG) in bladder cancer and their potential prognostic and therapeutic implications. We developed a SUMO pathway-related gene signature (SPRGS) prognostic model and revealed the potential of SPRGS as a predictive biomarker for chemotherapy response in bladder cancer. Patients in the SPRGS-Low subtype exhibited a higher response rate to chemotherapy and improved overall survival compared to those in the SPRGS-High subtype. Additionally, SPRGS showed promise in predicting the likelihood of response to immune checkpoint inhibitor therapy, suggesting its potential as a tool for personalized immunotherapy strategies in bladder cancer. We also identified EGR2 as a key gene associated with bladder cancer development and progression. Knockdown of EGR2 inhibited proliferation, invasion, and migration of bladder cancer cells, highlighting its functional role in the disease. Overall, this study provides a comprehensive analysis of the landscape of SPRG in bladder cancer and explores their potential prognostic and therapeutic implications. The findings contribute to a better understanding of the molecular characteristics of bladder cancer and may facilitate the development of personalized treatment strategies for bladder cancer patients.

## Figures and Tables

**Table 1:** The correlation between EGR2 protein expression and clinical characteristics of bladder cancer.

Characteristics	EGR2-Low expression	EGR2-Hihg expression	Total	*p*
	n=20	n=60	n=80	
**Age**				1.00
≤60	6	17	23	
>60	14	43	57	
**Sex**				0.43
Female	1	9	10	
Male	19	51	70	
**Grade**				0.13
Low	13	43	56	
High	7	17	24	
**Stage**				0.04
T1&2	16	30	46	
T3&4	4	30	34	

## References

[1] Dobruch J, Oszczudłowski M (2021). Bladder Cancer: Current Challenges and Future Directions. Medicina (Kaunas, Lithuania).

[2] Hu Y, Chen C, Tong X (2021). NSUN2 modified by SUMO-2/3 promotes gastric cancer progression and regulates mRNA m5C methylation. Cell death & disease.

[3] Yu L, Lin N, Ye Y (2024). The prognosis, chemotherapy and immunotherapy efficacy of the SUMOylation pathway signature and the role of UBA2 in lung adenocarcinoma. Aging.

[4] Yu L, Lin N, Ye Y (2024). Prognostic and chemotherapeutic response prediction by proliferation essential gene signature: Investigating POLE2 in bladder cancer progression and cisplatin resistance. Journal of Cancer.

[5] Wang Q, Zhang X, Chen L (2019). Regulation of the Expression of DAPK1 by SUMO Pathway. Biomolecules.

[6] Wang Q, Zhong W, Deng L (2021). The Expression and Prognostic Value of SUMO1-Activating Enzyme Subunit 1 and Its Potential Mechanism in Triple-Negative Breast Cancer. Frontiers in cell and developmental biology.

[7] Shi X, Du Y, Li S (2022). The Role of SUMO E3 Ligases in Signaling Pathway of Cancer Cells. International journal of molecular sciences.

[8] Ilic D, Magnussen HM, Tirard M (2022). Stress - Regulation of SUMO conjugation and of other Ubiquitin-Like Modifiers. Seminars in cell & developmental biology.

[9] Varejão N, Lascorz J, Li Y (2020). Molecular mechanisms in SUMO conjugation. Biochemical Society transactions.

[10] Sriramachandran AM, Dohmen RJ (2014). SUMO-targeted ubiquitin ligases. Biochimica et biophysica acta.

[11] Yang Y, He Y, Wang X (2017). Protein SUMOylation modification and its associations with disease. Open biology.

[12] Gong L, Qi R, Li DW (2017). Sumoylation Pathway as Potential Therapeutic Targets in Cancer. Current molecular medicine.

[13] Fan Y, Li X, Zhang L (2022). SUMOylation in Viral Replication and Antiviral Defense. Advanced science (Weinheim, Baden-Wurttemberg, Germany).

[14] Cheng J, Kang X, Zhang S (2007). SUMO-specific protease 1 is essential for stabilization of HIF1alpha during hypoxia. Cell.

[15] Lee JS, Chu IS, Heo J (2004). Classification and prediction of survival in hepatocellular carcinoma by gene expression profiling. Hepatology (Baltimore, Md).

[16] Shen HJ, Zhu HY, Yang C (2012). SENP2 regulates hepatocellular carcinoma cell growth by modulating the stability of β-catenin. Asian Pacific journal of cancer prevention: APJCP.

[17] Tan MY, Mu XY, Liu B (2013). SUMO-specific protease 2 suppresses cell migration and invasion through inhibiting the expression of MMP13 in bladder cancer cells. Cellular physiology and biochemistry: international journal of experimental cellular physiology, biochemistry, and pharmacology.

[18] Pei H, Chen L, Liao QM (2018). SUMO-specific protease 2 (SENP2) functions as a tumor suppressor in osteosarcoma via SOX9 degradation. Experimental and therapeutic medicine.

[19] Xie H, Gu Y, Wang W (2020). Silencing of SENP2 in Multiple Myeloma Induces Bortezomib Resistance by Activating NF-κB Through the Modulation of IκBα Sumoylation. Scientific reports.

[20] Chen S, Yu Z, Wang Y (2023). Block-Polymer-Restricted Sub-nanometer Pt Nanoclusters Nanozyme-Enhanced Immunoassay for Monitoring of Cardiac Troponin I. Analytical chemistry.

[21] Lin Q, Yu Z, Lu L (2023). Smartphone-based photoelectrochemical immunoassay of prostate-specific antigen based on Co-doped Bi(2)O(2)S nanosheets. Biosensors & bioelectronics.

[22] Zeng R, Qiu M, Wan Q (2022). Smartphone-Based Electrochemical Immunoassay for Point-of-Care Detection of SARS-CoV-2 Nucleocapsid Protein. Analytical chemistry.

[23] Yin M, Joshi M, Meijer RP (2016). Neoadjuvant Chemotherapy for Muscle-Invasive Bladder Cancer: A Systematic Review and Two-Step Meta-Analysis. The oncologist.

[24] Neoadjuvant chemotherapy in invasive bladder cancer (2005). update of a systematic review and meta-analysis of individual patient data advanced bladder cancer (ABC) meta-analysis collaboration. European urology.

[25] Winoker JS, Liaw CW, Galsky MD (2020). Clinical Complete Response after Neoadjuvant Chemotherapy for Muscle-invasive Bladder Cancer: A Call for Standardized Assessments and Definitions. European urology focus.

[26] Del Bene G, Sternberg CN (2017). Systemic chemotherapy in muscle invasive and metastatic bladder cancer: present and future. Urologia.

[27] Xu T, Xu W, Zheng Y (2022). Comprehensive FGFR3 alteration-related transcriptomic characterization is involved in immune infiltration and correlated with prognosis and immunotherapy response of bladder cancer. Frontiers in immunology.

[28] Brancolini C, Gagliano T, Minisini M (2022). HDACs and the epigenetic plasticity of cancer cells: Target the complexity. Pharmacology & therapeutics.

[29] Cuttini E, Goi C, Pellarin E (2023). HDAC4 in cancer: A multitasking platform to drive not only epigenetic modifications. Frontiers in molecular biosciences.

[30] Hatakeyama S (2017). TRIM Family Proteins: Roles in Autophagy, Immunity, and Carcinogenesis. Trends in biochemical sciences.

[31] Liu S, Tian Y, Zheng Y (2020). TRIM27 acts as an oncogene and regulates cell proliferation and metastasis in non-small cell lung cancer through SIX3-β-catenin signaling. Aging.

[32] Xing L, Tang X, Wu K (2020). TRIM27 Functions as a Novel Oncogene in Non-Triple-Negative Breast Cancer by Blocking Cellular Senescence through p21 Ubiquitination. Molecular therapy Nucleic acids.

[33] Sakamoto T, Kuboki S, Furukawa K (2023). TRIM27-USP7 complex promotes tumour progression via STAT3 activation in human hepatocellular carcinoma. Liver international: official journal of the International Association for the Study of the Liver.

[34] Young E, Noerenberg D, Mansouri L (2017). EGR2 mutations define a new clinically aggressive subgroup of chronic lymphocytic leukemia. Leukemia.

[35] Oakes CC, Seifert M, Assenov Y (2016). DNA methylation dynamics during B cell maturation underlie a continuum of disease phenotypes in chronic lymphocytic leukemia. Nature genetics.

[36] Damm F, Mylonas E, Cosson A (2014). Acquired initiating mutations in early hematopoietic cells of CLL patients. Cancer discovery.

[37] Ying Y, Ma X, Fang J (2021). EGR2-mediated regulation of m(6)A reader IGF2BP proteins drive RCC tumorigenesis and metastasis via enhancing S1PR3 mRNA stabilization. Cell death & disease.

[38] Watanabe TK, Fujiwara T, Kawai A (1996). Cloning, expression, and mapping of UBE2I, a novel gene encoding a human homologue of yeast ubiquitin-conjugating enzymes which are critical for regulating the cell cycle. Cytogenetics and cell genetics.

[39] Li F, Lai L, You Z (2022). Identification of UBE2I as a Novel Biomarker in ccRCC Based on a Large-Scale CRISPR-Cas9 Screening Database and Immunohistochemistry. Frontiers in molecular biosciences.

[40] Chen SF, Gong C, Luo M (2011). Ubc9 expression predicts chemoresistance in breast cancer. Chinese journal of cancer.

[41] Zhao Z, Tan X, Zhao A (2012). microRNA-214-mediated UBC9 expression in glioma. BMB reports.

[42] Wang S, Jiao B, Geng S (2014). Combined aberrant expression of microRNA-214 and UBC9 is an independent unfavorable prognostic factor for patients with gliomas. Medical oncology (Northwood, London, England).

[43] Yang H, Gao S, Chen J (2020). UBE2I promotes metastasis and correlates with poor prognosis in hepatocellular carcinoma. Cancer cell international.

[44] Montazeri K, Bellmunt J (2020). Erdafitinib for the treatment of metastatic bladder cancer. Expert review of clinical pharmacology.

